# Systematic and historical biogeography of the Bryconidae (Ostariophysi: Characiformes) suggesting a new rearrangement of its genera and an old origin of Mesoamerican ichthyofauna

**DOI:** 10.1186/1471-2148-14-152

**Published:** 2014-07-08

**Authors:** Kelly T Abe, Tatiane C Mariguela, Gleisy S Avelino, Fausto Foresti, Claudio Oliveira

**Affiliations:** 1 Departamento Morfologia, Instituto de Biociências, UNESP – Universidade Estadual Paulista, Botucatu, São Paulo, Brazil

**Keywords:** Characiformes, Bryconidae, Evolution, Phylogeny, Systematics

## Abstract

**Background:**

Recent molecular hypotheses suggest that some traditional suprageneric taxa of Characiformes require revision, as they may not constitute monophyletic groups. This is the case for the Bryconidae. Various studies have proposed that this family (considered a subfamily by some authors) may be composed of different genera. However, until now, no phylogenetic study of all putative genera has been conducted.

**Results:**

In the present study, we analyzed 27 species (46 specimens) of all currently recognized genera of the Bryconidae (ingroup) and 208 species representing all other families and most genera of the Characiformes (outgroup). Five genes were sequenced: 16SrRNA, Cytochrome *b*, recombination activating gene 1 and 2 and myosin heavy chain 6 cardiac muscle. The final matrix contained 4699 bp and was analyzed by maximum likelihood, maximum parsimony and Bayesian analyses. The results show that the Bryconidae, composed of *Brycon*, *Chilobrycon*, *Henochilus* and *Salminus*, is monophyletic and is the sister group of Gasteropelecidae + Triportheidae. However, the genus *Brycon* is polyphyletic. Fossil studies suggest that the family originated approximately 47 million years ago (Ma) and that one of the two main lineages persisted only in trans-Andean rivers, including Central American rivers, suggesting a much older origin of Mesoamerican ichthyofauna than previously accepted.

**Conclusion:**

Bryconidae is composed by five main clades, including the genera *Brycon*, *Chilobrycon*, *Henochilus* and *Salminus*, but a taxonomic review of these groups is needed. Our results point to a possible ancient invasion of Central America, dating about 20.3 ± 5.0 Ma (late Oligocene - early Miocene), to explain the occurrence of *Brycon* in Central America.

## Background

The order Characiformes contains approximately 2000 species distributed among 23 families, with 19 exclusively Neotropical families and four exclusively African families [1-3]. The order comprises one of the largest freshwater fish radiations. Among characiform genera, *Brycon* is one of the most speciose, containing 42 species [4,5]. Members of this genus occur from southern Mexico to Panama, across the trans-Andean South American river basins from northern Peru to the Maracaibo system in Venezuela, in all major river drainages in cis-Andean South America, and in most Atlantic and Caribbean coastal river basins [4]. *Brycon* species are medium- to large-sized fishes, with a maximum standard length from 15 cm (*Brycon pesu*) to approximately 70 cm (*Brycon orbygnianus* and *Brycon amazonicus*). *Brycon* species are important food fishes throughout Central and South America [4], with catches estimated to approximately 5,100 tons for the year 2007 in Brazil [6].

Despite their wide distribution, species diversity, and commercial importance, the taxonomy of the Bryconidae remains unclear. Species in Panama and the trans-Andean rivers of northern South America were extensively studied by Eigenmann [7], Hildebrand [8] and Dahl [9]. In contrast, the taxonomy of *Brycon* species in the cis-Andean river basins was revised by Lima [10] in an unpublished master's thesis. Lima [4] published a taxonomic synthesis of the Bryconinae, which, in addition to the genus *Brycon*, included two monotypic genera: *Chilobrycon* Géry & Rham, 1981 and *Henochilus* Garman, 1890.

The phylogenetic relationships within the Bryconidae have been the subject of several studies; however, a detailed hypothesis of the relationships among its species and with other Characiformes is absent. Regan [11] was the first author to propose a relationship between *Brycon* and *Chalceus*. In the same study, he further proposed that *Salminus* and *Hystricodon* (=*Exodon*) were related to *Brycon*. Eigenmann [12] proposed the classification of the subfamily Bryconinae, including the genera *Brycon* and *Chalceus*. Géry [13] proposed that the Bryconinae instead be classified as the subfamily Chalceinae; however, in 1972, Géry [14] followed the Bryconinae classification and divided the subfamily in three tribes: Bryconini, Triportheini and Salminini. The same classification scheme was used in Géry´s [15] seminal book on characiforms.

In a comparative osteological study of *Brycon* and *Salminus*, Roberts [16] suggested that the apparent similarities between them may reflect the primitive position of *Brycon*. Uj [17] proposed a new classification, the family Bryconidae, which included *Brycon*, *Chalceus, Catabasis*, *Lignobrycon*, *Salminus*, *Triportheus*, *Chilobrycon* and *Bryconexodon*. Mirande [18] recognized the subfamily Bryconinae, comprised of *Brycon*, *Triportheus*, *Chilobrycon*, *Henochilus*, and *Lignobrycon*. The latter three genera were not studied by Mirande [18]; however, whereas *Chilobrycon* and *Henochilus* are recognized as closely related to *Brycon*[4], *Lignobrycon* appears distinct, and the absence of representatives of this genus may be responsible for the unusual result reported by the author. Molecular data [1,19-21] supports the close relationship between *Brycon* and *Salminus* proposed by Uj [17]. In the broadest molecular phylogenetic study of the Characiformes published to date, Oliveira *et al.*[1] analyzed specimens of *Brycon*, *Henochilus* and *Salminus* and recognized them as a monophyletic group, the family Bryconidae.

Given the importance of the Bryconidae among the Characiformes, as stated above, two mitochondrial and three nuclear genes of representatives of all genera of this family, along with representatives of all other Characiformes families [as defined by 1], were analyzed in the present study to formulate a hypothesis of the relationships among species and genera of the Bryconidae and between this family and the other Characiformes. In addition, a time-calibrated tree was constructed to investigate the temporal relationships between the origin of Bryconidae groups and the main geological events in South America.

## Methods

### Selection of taxa and delineation of the ingroup and outgroup

**Figure 1:**
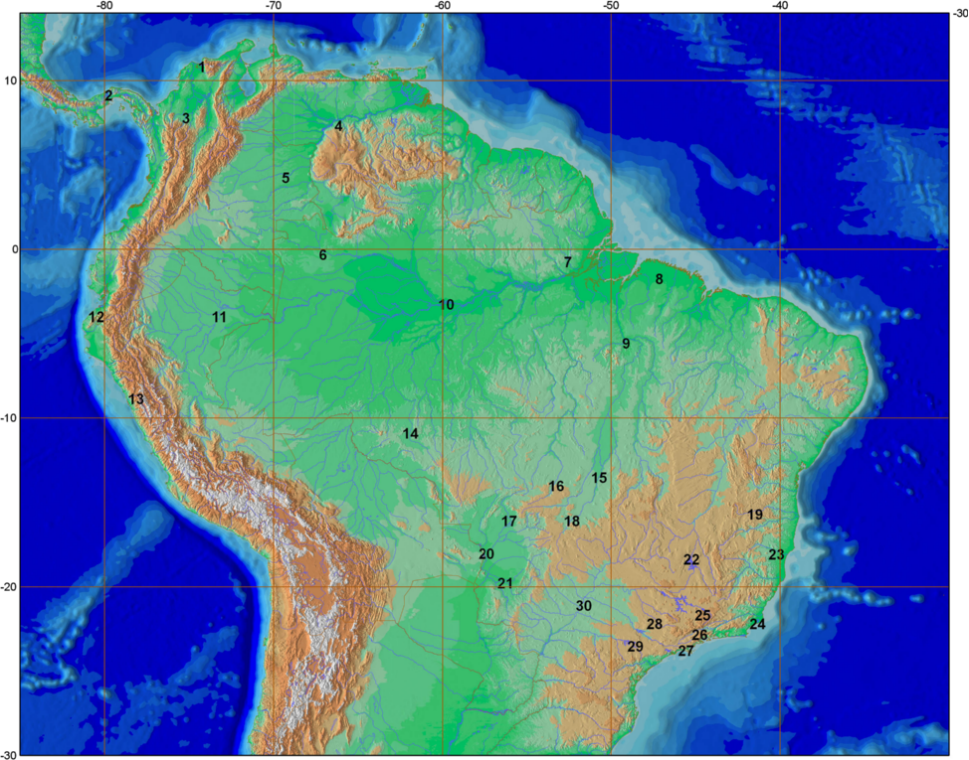
**Geographical distribution of the Bryconidae samples.** 1-*Brycon moorei*; 2-*Brycon chagrensis*, *Brycon petrosus*; 3-*Brycon moorei*, *Salminus affinis*; 4-*Brycon amazonicus*, *Brycon falcatus*; 5-*Brycon amazonicus*; 6-*Brycon falcatus*; 7-*Brycon pesu*; 8-*Brycon pesu*; 9-*Salminus sp.*; 10-*Brycon amazonicus*; 11-*Brycon melanopterus*; 12-*Chilobrycon deuterodon*; 13-*Brycon* aff. *atrocaudatus*; 14-*Brycon falcatus*; 15-*Brycon gouldingi*; 16-*Brycon* cf. falcatus; 17-*Brycon hilarii*; 18-*Brycon pesu*; 19-*Brycon sp.*; 20-*Brycon hilarii*; 21-*Brycon hilarii*; 22-*Brycon orthotaenia*, *Salminus franciscanus*; 23-*Brycon ferox, Brycon vermelha*, *Henochilus wheatlandii*; 24-*Brycon insignis*; 25-*Brycon nattereri*; 26-*Brycon opalinus*; 27-*Brycon opalinus*; 28-*Brycon orbignyanus*, *Salminus brasiliensis*; 29-*Salminus hilarii*; 30-*Brycon nattereri*. Map constructed with the program QGIS 2.2.0 (http://www.qgis.org) using layers obtained in the websites http://www.earthobservatory.nasa.gov and http://www.ibge.gov.br/home/geociencias.

The ingroup was composed of 46 specimens including 27 species of all four recognized genera of the Bryconidae (Table 1, Figure 1). To replace the Bryconidae into the evolutionary tree of the Characiformes we used the matrix employed by Oliveira *et al.*[1] in their broad study of Characidae relationships, including 208 samples representing all Characiformes families (Additional file 1). All specimens for this study were collected in accordance with Brazilian laws under a permanent scientific collection license in the name of Dr. Claudio Oliveira (IBAMA-SISBIO, 13843-1). Additionally, this survey was carried out in strict accordance with the recommendations for the National Council for Control of Animal Experimentation and Federal Board of Veterinary Medicine. The studied material was deposited in the Laboratório de Biologia e Genética de Peixes (LBP), Instituto de Biociências, Universidade Estadual Paulista, Botucatu, Sao Paulo, Brazil.

**Table 1:** Species of Bryconidae analyzed in the present phylogenetic study

**Species**	**Voucher**	**Specimen**	**Locality**	**Geographic position**	**Position in Figure **1
*Brycon amazonicus*	2187	15565	Laguna de Castilleros, Venezuela	07º30'50.9''N 66º09'19.8'' W	4
		15567			
*Brycon amazonicus*	2859	18988	Rio Tomo, Colombia	04°25'27.1'' N 69°17'12.5'' W	5
*Brycon amazonicus*	834	8835	Rio Negro, Amazonas, Brazil	03°05'05.2''.S 59°47'23.7'' W	10
*Brycon* aff. *atrocaudatus*	1356	17096	Rio Santa, Peru	08°40’24.0'' S 78°09'16.3'' W	13
*Brycon chagrensis*	2749	18510	Río Llano Sucio, Panama	09°19’26.2'' N 79°46'08.2'' W	2
*Brycon falcatus*	2668	15563	Laguna de Castilleros, Venezuela	07º30’50.9” N 66º09’19.8” W	4
*Brycon falcatus*	5146	26278	Rio Machado, Rondônia, Brazil	10°43'36.0' S 61°55'12.9'' W	14
*Brycon falcatus*	6878	32395	Rio Negro, Amazonas, Brazil	00°08'09.4" S 67°05'03.4" W	6
*Brycon* cf. *falcatus*	8109	37580	Rio Culuene, Mato Grosso, Brazil	13°49'00.0'' N 53°15'08.0'' W	16
		37581			
*Brycon ferox*	2855	18979	Aquaculture	-	-
*Brycon ferox*	8099	37528	Rio Mucuri, Minas Gerais, Brazil	17°41'42.4' S 40°46'11.3'' W	23
*Brycon ferox*	8100	37529	Rio Mucuri, Minas Gerais, Brazil	17°41'42.4' S 40°46'11.3'' W	23
*Brycon gouldingi*	3130	19203	Lagoa da Égua, Mato Grosso, Brazil	13°20'05.1'' S 50°42'16.2'' W	15
*Brycon henni*	2857	18984	Colombia	Aquaculture	-
*Brycon hilarii*	3805	21895	Rio Negro, Mato Grosso do Sul, Brazil	19°34'33.7' S 56°14'49.5'' W	21
*Brycon hilarii*	2766	17634	Rio Cuiabá, Rio São Lourenço, Mato Grosso, Brazil	17°50’45.3'' S 57°24'11.7'' W	20
*Brycon hilarii*	4676	24810	Rio Cuiabá, Mato Grosso, Brazil	15°54'50.0’ S 56°02'07.0' W	17
*Brycon insignis*	2309	16075	Lagoa Feia, Rio de Janeiro, Brazil	22°00'00.0'' S 41°20'00.0'' W	24
*Brycon melanopterus*	9778	38096	Rio Amazonas, Iquitos, Peru	03°48'11.5' S 73°13'12.4'' W	11
		38097			
*Brycon moorei*	2858	18986	Rio Rancheria, Colombia	11°0' 23.57 N 74°14'48.80'' W	1
*Brycon moorei*	12817	55010	Rio Cauca, Antioquia, Colombia	07°57'28.5'' N 75°12'00.0'' W	3
*Brycon nattereri*	2856	18981	Rio Paraná, São Paulo, Brazil	20°55'27.90'' S 51°37'32.62'' W	30
		18982			
*Brycon nattereri*	8101	37541	Rio Capivari, Minas Gerais, Brazil	21° 30' 16.0" S 44°34' 29" W	25
*Brycon opalinus*	6303	29001	Rio Itagaçaba, São Paulo, Brazil	22°39'26.3'' S 44°45'49.8'' W	26
*Brycon opalinus*	6306	29349	Rio dos Prazeres, São Paulo, Brazil	23°35'43.8'' S 45°34'08.0'' W	27
*Brycon orbignyanus*	2746	18004	Aquaculture, Brazil	21°59'45.74'' S 47°25'36.57' W'	28
*Brycon orthotaenia*	249	4215	Rio São Francisco, Minas Gerais, Brazil	18°11'28.50" S 45°14'51.42" W	22
*Brycon pesu*	8111	37578	Rio das Garças, Mato Grosso, Brazil	15°54'18.1'' S 52°19'24.2'' W	18
		37579			
*Brycon pesu*	5320	26930	Rio Jari, Amapá, Brazil	00°34’11” S 52°33’19'' W	7
*Brycon pesu*	9409	42567	Rio Guamá, Pará, Brazil	01°34'00.5'' S 47°09'51.4'' W	8
*Brycon petrosus*	2750	18504	Río Llano Sucio, Panama	09°19’26.2'' N 79°46'08.2'' W	2
*Brycon vermelha*	9066	42508	Rio Mucuri, Minas Gerais, Brazil	17°41'42.4' S 40°46'11.3'' W	23
*Brycon sp.*	5837	28350	Estação de Piscicultura da CEMIG, Minas Gerais, Brazil	15°31'19.0'' S 41°30'18.0'' W	19
*Chilobrycon deuterodon*	9334	45001	Rio Tumbes, Peru	03°48'17.9'' S 80°29'52.5'' W	12
		45002			
*Henochilus wheatlandii*	1221	25846	Rio Santo Antônio, Minas Gerais, Brazil	17°53 60.00'' S 40° 13'0.00'' W	23
*Salminus affinis*	12817	55009	Rio Cauca, Antioquia, Colombia	07°57'28.5'' N 75°12'00.0'' W	3
*Salminus brasiliensis*	850	9025	Rio Mogi-Guaçu, São Paulo, Brazil	21°55' 37.60'' S 47°22'4.40'' W	28
*Salminus franciscanus*	8090	37503	Rio São Francisco, Minas Gerais, Brazil	18°11'21.0' S 45°15'10.3'' W	22
*Salminus hilarii*	84	7615	Rio Paranapanema, São Paulo, Brazil	23°20' S 48°34' W	29
*Salminus sp.*	8160	38065	Rio Tapirapé, Pará, Brazil	05° 22' 22.30'' S 49°07'0.94'' W	9

Asterisks indicate specimens sequenced in Oliveira *et al. *[1].

### Molecular data collection

Total DNA was extracted from ethanol-preserved muscle samples using the DNeasy Tissue Extraction Kit (Qiagen), following the manufacturer’s instructions. Partial sequences of the mitochondrial genes 16SrRNA and Cytochrome *b* (Cytb) and the nuclear genes recombination activating gene 1 (Rag1), recombination activating gene 2 (Rag2) and myosin, heavy chain 6, cardiac muscle, alpha (Myh6) were amplified by polymerase chain reaction (PCR) with the same primers utilized by Oliveira *et al.*[1]. Amplifications were performed in a total volume of 25 μl consisting of 2.5 μl of 10X buffer (10 mM Tris-HCL + 15 mM MgCl2 buffer), 0.5 μl of MgCl_2_ (50 mM), 0.5 μl of each primer (5 μM); 0.4 μl of dNTPs (200 nM of each), 0.2 μl of Taq Platinum polymerase (Invitrogen; 5 U/μl), 1 μl of template DNA (10-50 ng) and 19.4 μl of ddH_2_O. The thermo-cycler profile used for the fragments 16SrRNA and Cyt *b* consisted of 35 cycles, 30 s at 95°C, 45-120 s at 50-55°C (according to primer and species), and 90 s at 72°C. Nested-PCR was used to amplify the nuclear genes Rag1, Rag2 and Myh6. Amplification conditions for these genes in both rounds of PCR consisted of 15 cycles, 30 s at 95°C, 45 s at 56°C (according to primer), and 30 s at 72°C followed by 15 cycles, 30 s at 95°C, 45 s at 54°C (according to primer), and 90 s at 72°C. PCR products were purified using ExoSap-IT® (USB Corporation), sequenced using the “Big DyeTM Terminator v 3.1 Cycle Sequencing Ready Reaction Kit” (Applied Biosystems), purified again by ethanol precipitation and loaded into an automatic sequencer 3130-Genetic Analyzer (Applied Biosystems) at Instituto de Biociências, Universidade Estadual Paulista, Botucatu, São Paulo, Brazil. Contigs were assembled and edited in BioEdit 7.0.9.0 [22]. In cases of unclear nucleotide identity, IUPAC ambiguity codes were applied. All obtained sequences were deposited in GenBank (Table 1).

### Alignment and phylogenetic analyses

Sequences of each gene were aligned using the Muscle algorithm under default parameters [23] and the alignments inspected by eye for any obvious misalignments that were subsequently corrected. A quality control step was included in our workflow as described in Oliveira *et al.*[1]. Genetic distances among sequences were calculated in Mega 5.04 [24]. To evaluate the occurrence of substitution saturation, we estimated the index of substitution saturation (Iss) in DAMBE 5.2.31 [25] as described in Xia *et al.*[26] and Xia and Lemey [27].

A set of six reasonable partitioning schemes, ranging from 1 to 13 partitions (Table 2), was tested following the procedures outlined by Li *et al.*[28] using the AIC and BIC. The best-fit model of nucleotide substitution was searched in Mega 5.04 [24] under default parameters using the Akaike information criterion (see [29], for justification).

**Table 2:** Comparison of log likelihoods, AIC and BIC values among different partitioning schemes (from 1 to 13 partitions)

Number of partitions*	**Number of parameters**	**L**_***ML***_	**AIC**	**Delta**_***i***_	**BIC**_***ML***_
1	9	181065.424	362148.847	9956.687	362163.896
2	19	179598.394	359234.787	7042.627	359266.555
4A	39	180039.825	360157.651	7965.491	360222.859
4B	39	179526.116	359130.233	6938.073	359195.441
5	49	179380.393	358858.787	6666.627	358940.715
13	129	175967.080	352192.160	0.000	352407.849

*1 partition = all datasets; 2 partitions = mitochondrial (16S + CytB) and nuclear (Myh6 + Rag1 + Rag2); 4 partitions A = 16S and 1st, 2nd, and 3rd codon position of protein coding genes; 4 partitions B = 16S + CytB and 1st, 2nd, and 3rd codon position of nuclear genes; 5 partitions = by each gene (16S + CytB + Myh6 + Rag1 + Rag2); 13 partitions = 16S + each codon position of each protein coding genes (1st, 2nd, and 3rd codon position of CytB; 1st, 2nd, and 3rd codon position of Myh6; 1st, 2nd, and 3rd codon position of Rag1; 1st, 2nd, and 3rd codon position of Rag2).

For each type of analysis, the following results are shown: total number of parameters, log likelihood calculated using RAxML (*L*_*ML*_), AIC values, the difference in AIC values among model *i* and the best model (Δ*i* = AIC*i* – AICmin), BIC_*ML*_ values.

Maximum parsimony (MP) analyses were conducted with PAUP* 4.0b10 [30]. Heuristic searches were performed with minimally 1000 random addition replicates and TBR branch swapping. All characters were unordered, all character transformations were equally weighted, and branches with a maximum length of zero were collapsed. Gaps were treated as missing data since experiments were they were treated as a fifth base did not result in better resolved trees. Clade robustness was assessed using 1000 bootstrap pseudoreplicates [31] with the same parameters as described above.

RAxML [32], running in the web servers RAxML-HPC2 on TG [33,34], was used for all maximum likelihood analyses with a mixed partition model. Random starting trees were ran for each independent ML tree search, and all other parameters were set to default values. All ML analyses were conducted following the 13 partitions sch. as suggested by the AIC and BIC (Table 2). Topological robustness was investigated using 1000 non-parametric bootstrap replicates.

Phylogenetic analyses using a partitioned Bayesian inference were conducted in MrBayes 3.1.2 [35]. A mixed model analysis was implemented, allowing individual models of nucleotide substitution to be estimated independently for each partition. Because MrBayes 3.1.2 only implements 1, 2, and 6 substitution rate models, it was often not possible to implement the preferred model as selected by the AIC. In these situations, the nearest overparameterized model was used to avoid the negative consequences of model violation or underparameterization [28,36]. As a consequence, the model for all partitions was set as “lset nst = 6” and “rates = invgamma” (G + I), with the commands “unlink” and “prset ratepr = variable” used to unlink the model parameters across the data partitions and define a rate multiplier for each partition. Two independent Bayesian analyses were conducted. Four independent MCMC chains were run with 30,000,000 replicates each, with one tree sampled every 1000 steps. The distribution of the log likelihood scores was examined to determine stationarity for each search and decide if extra runs were required to achieve convergence using the program Tracer 1.4 [37]. Initial trees estimated prior to convergence were discarded as part of the burn-in procedure, and the remaining trees were used to construct a 50% majority rule consensus tree in PAUP*.

The estimation of divergence times in the inferred phylogeny was carried out using BEAST (Bayesian evolutionary analysis sampling trees) 1.8.0 [38] on a reduced dataset that included the family Bryconidae and representatives of the families Gasteropelecidae and Triportheidae. To calibrate our molecular tree we followed the guideline proposed by Parham et al. [39]. Initially, two fossils were chosen: *Lignobrycon ligniticus* and *Brycon avus. Lignobrycon ligniticus* (Woodward, 1898) (type specimen: BMNH P9012) was described in the genus *Tetragonopterus* and moved to *Lignobrycon* by Eigenmann and Myers [40]. Malabarba [41] in a phylogenetic study showed that *L. ligniticus* is the sister group of *L. myersi* (a species included in our phylogeny) and these two species are the sister group of *Triportheus. Brycon avus* (Woodward, 1898) (type specimen: BMNH P9224) was described in the genus *Tetragonopterus* and moved to *Brycon* by Travassos and Silva [42]. Malabarba ([43] – unpublished thesis) showed that *B. avus* is placed within the genus *Brycon* but its relationships with the remaining species of this genus was not resolved which make very difficult its use in our phylogeny. Considering that *B. avus* does not meet all criteria proposed by Parham et al. [39] we discuss it putative relationship with the species we analyzed in the present study but we did not use it to calibrate our trees.

These two species were described based on complete specimens collected in the Tremembé Formation, Taubaté Basin, São Paulo, Brazil. Geological studies [44] have confirmed the age of this formation as Oligocene, as also suggested by studies in mammalian fossils [45] and pollens [46,47]. According to the International Commission on Stratigraphy (http://www.stratigraphy.org) Oligocene extended from 33.9 to 23.03 million years ago (Ma). These dates were implemented in BEAST with a log-normal prior offset with a mean and standard deviation of 28.5 ± 5.5. We used a birth-death model for the speciation likelihood and a random starting tree. The analysis was run for 50 million generations and sampled every 10000^th^ generation. Stationarity and sufficient mixing of parameters (ESS > 200) were checked using Tracer 1.5 [47]. A consensus tree was built using TreeAnnotator v 1.6.2 [48].

## Results

Partial sequences of two mitochondrial (16SrRNA and Cytb) and three nuclear genes (Myh6, Rag1 and Rag2) were obtained for 254 specimens, 41 of which were sequenced in the present study (Additional file 1). The final matrix contained 4699 bp and was deposited in TreeBase (http://www.treebase.org) under number 15409 and in DRYAD (http://www.datadryad.org - http://datadryad.org/resource/doi:10.5061/dryad.kt24p).

Missing data due to problems with the PCR, sequencing problems, or missing data in GenBank corresponded to 11.4% of the matrix (Table 3). Data absence was more prevalent among nuclear (15.7%) than mitochondrial genes (4.9%), perhaps due to non-conserved priming regions and a higher risk of cross-contamination in the nested PCR procedure. For each matrix and gene, the number and percentage of sequences obtained, their size (bp), the number of variable sites, their base pair composition, the overall mean genetic distance (p-distance), the best substitution model for the gene, the α (shape) parameter of Γ distribution, the proportion of invariant (I) sites, the number of informative characters under parsimony, and the proportion of informative characters under parsimony are presented in Table 3. Under the MP criterion, 53.2% of the positions were phylogenetically informative. The overall mean genetic distance observed was between 0.083 ± 0.004 (Myh6) and 0.216 ± 0.007 (CytB), suggesting that the analyzed sequences contain sufficient genetic variation for an informative phylogenetic study of species, genera and families. Each gene and codon position partition was further tested to investigate the occurrence of substitution saturation [26,27]. The results showed significant saturation for only the 3rd codon position of Cytb in the symmetrical topology test (results not shown); however, considering that the Iss.c value is greater than the Iss value and that there is no significant saturation in the asymmetrical topology test, the information found at this position can be used in the phylogenetic analysis [26,27]. The best-fitting model of nucleotide substitution calculated for each gene was: GTR + I + Γ (16S), TN93 + I + Γ (CytB), T92 + I + Γ (Myh6) and K2P + I + Γ (Rag1, Rag2) (Table 3). The combined data set contains significant phylogenetic information, as most major lineages along the backbone of the tree were supported by high bootstrap values.

**Table 3:** Information content and characteristics of each gene partition

			**Gene**			
	**16S**	**CytB**	**Myh6**	**Rag1**	**Rag2**	**Total**
Number of sequences	254 (100%)	229 (90.2%)	215 (84.6%)	207 (81.5%)	220 (86.6%)	254
bp after alignment	653	992	755	1265	1034	4699
Number of variable sites	393	684	392	874	685	3028
Number of informative characters under parsimony	326	585	327	680	581	2499
% informative characters under parsimony	49.9	58.9	43.3	51.4	56.2	53.2
*Π*_T_	0.227	0.296	0.246	0.226	0.231	0.245
*Π*_C_	0.236	0.293	0.214	0.240	0.252	0.249
*Π*_A_	0.313	0.265	0.307	0.252	0.245	0.271
*Π*_G_	0.224	0.146	0.234	0.283	0.272	0.234
Overall mean genetic distance (p-distance)	0.122 ± 0.008	0.216 ± 0.007	0.083 ± 0.004	0.108 ± 0.004	0.110 ± 0.004	0.134 ± 0.002
Nucleotide substitution model	GTR + I + Γ	TN93 + I + Γ	T92 + I + Γ	K2P + I + Γ	K2P + I + Γ	GTR + I + Γ
α (shape) parameter of Γ distribution	0.65	0.65	1.03	0.89	0.97	0.61
Proportion of invariants (I) sites	0.32	0.29	0.42	0.22	0.26	0.29

Six different partitioning schemes, ranging from one to 13 partitions (Table 2), were tested to establish the optimal number of data partitions (following [49]) for the final analysis. The results showed that the 13 partition model was the best choice; however, ML analysis conducted with the other partitioning schemes produced the same final topology, with minor differences in branch length and support values (data not shown).

**Figure 2:**
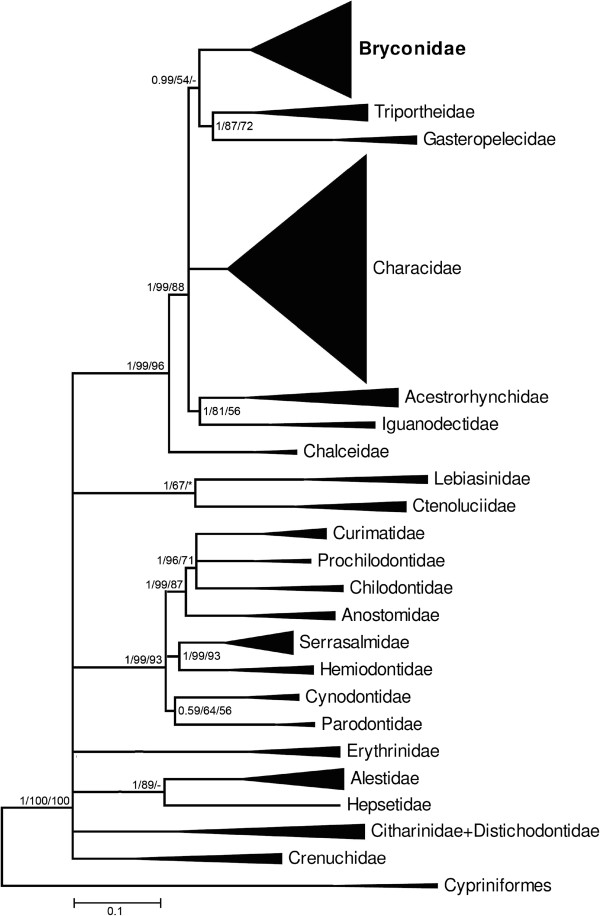
**Summary tree showing relationships among major lineages obtained by a maximum likelihood (ML) partitioned analysis of the concatenated dataset and emphasizing the relationships among species of Bryconidae (bold).** A series of three numbers (e.g., 1/100/87) at each of the main nodes represents the posterior probability for that split obtained in the Bayesian analysis (B), percentage of bootstrap support obtained by ML analysis, and percentage of bootstrap support obtained by MP analysis, respectively (1000 bootstrap replicates). Dashes represent values less than 0.5 (B) or 50% (ML, MP). Asterisks represent nodes that were not obtained by B or MP analyses.

Throughout the text and in the figures, measures of support are represented by a series of three numbers on selected internal branches of the trees subtending labeled clades, with the first number indicating the posterior probabilities from the Bayesian analysis (B) and the following numbers indicating the non-parametric bootstrap percentages from the ML and MP analyses, respectively (e.g., 1/100/100; see Figure 2). Dashes represent values lower than 0.5 (B) or 50% (ML, MP), and asterisks represent nodes with varying topologies depending on the analytical method employed. Nodes without support values greater than 0.5 (B) and 50% (ML, MP) were collapsed. A ML tree summarizing the phylogenetic results is presented in Figure 2. The same tree expanded to show all taxa is presented in the Additional file 2. The general tree topology observed in all analyses was very similar, although statistical support was weak at some nodes. Thus, we choose the Bayesian topology obtained with BEAST to discuss the relationships among taxa and we present the differences among this result and those obtained with other techniques in the text where appropriated.

### Phylogenetic relationships of the Bryconidae

**Figure 3:**
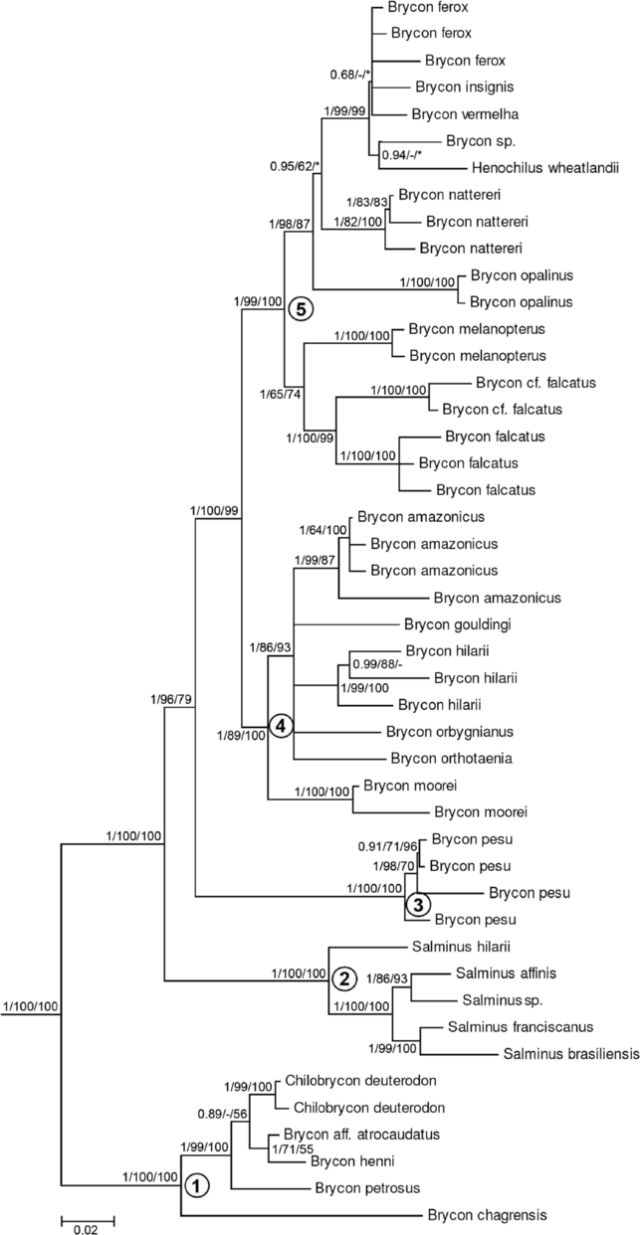
**Best maximum likelihood (ML) tree of the Bryconidae obtained in the partitioned analysis of the concatenated dataset.** Numbered nodes as referenced in text and values shown in Figure 2. Numbers after species names, between braches, refer to collecting sites shown in the Figure 1; dashes represent fishes from aquaculture without known locality.

As shown in Figure 3, Bryconidae is monophyletic with very strong statistical support (1/100/100). Bryconidae appears as the sister group of the Gasteropelecidae + Triportheidae in all analyses but the support in MP studies was less than 50% (0.99/54/-) (Figure 2). Within the Bryconidae, we identified five clades and the genus *Brycon* turned out as polyphyletic (Figure 3). The first clade (1/100/100) is composed of some trans-Andean species of *Brycon* and *Chilobrycon*. The second clade (1/100/100) is composed entirely of *Salminus*. The third clade (1/100/100) is comprised of four samples of *Brycon pesu*. The fourth clade (1/89/100) is composed of one trans-Andean species of *Brycon* and additional *Brycon* species from the Amazon, Orinoco, São Francisco, and Paraná-Paraguay basins. The fifth clade (1/99/100) is composed of some *Brycon* from the Amazon and Paraná basins, *Brycon* from the Brazilian coast and *Henochilus*.

### Estimates of divergence times of Bryconidae clades

**Figure 4:**
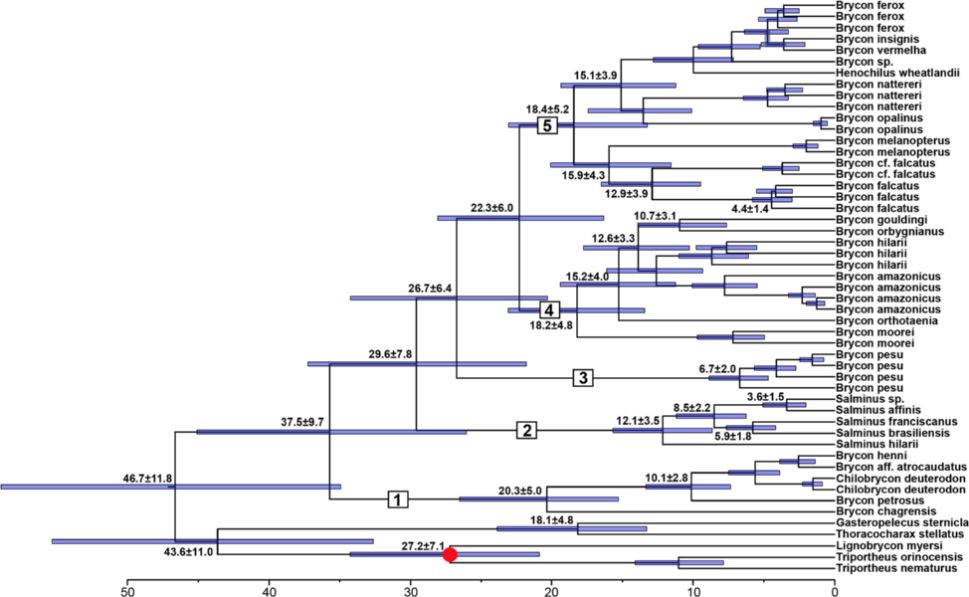
**The BEAST chronogram tree from 50 million generations, indicating the divergence over time of the family Bryconidae.** Red circle shows the calibration points based on the fossil *Lignobrycon ligniticus* (28.5 ± 5.5 Ma). Scale = millions of years before present.

Using the fossil of *Lignobrycon ligniticus* to calibrate our phylogenetic tree we found that the mean substitution rate for the Bryconidae dataset, estimated using BEAST, was 0.001847% per Ma. The origin of the Bryconidae, calculated according the available fossil information described above, was estimated at 46.7 Ma (95% HPD: 34.9 – 58.9) (Figure 4). Within the Bryconidae, clade 1 originated 35.7 Ma (95% HPD: 26.1 – 45.1), clade 2 originated 29.6 Ma (95% HPD: 21.8 - 37.2), clade 3 originated 26.7 Ma (95% HPD: 20.1 – 34.2) and clades 4 and 5 both originated 22.3 Ma (95% HPD: 16.3 - 28.1).

## Discussion

### Phylogenetic relationships among the Bryconidae and other Characiformes taxa

Our study represents the first phylogenetic analysis in which all genera currently assigned to the Bryconidae [1,4] were investigated. Our results indicate that *Brycon*, *Chilobrycon* and *Henochilus* belong to a monophyletic group, as suggested by Lima [4]. Our study also corroborates the hypothesis that *Salminus* is closely related to *Brycon*[1,17,20,21,50,51].

All of our analyses identified the Bryconidae as the sister group of the clade composed of the families Gasteropelecidae and Triportheidae This is an interesting result, as our previous study [1] suggested that the Bryconidae may be the sister group of the Gasteropelecidae. These different results appear to be due to the larger number of representatives of the Bryconidae in the present analysis, emphasizing the importance of using a large number of taxa in phylogenetic studies.

According to Weitzman [52], the presence of expanded coracoids in *Triportheus* and the so-called subfamily Gasteropelecinae is likely due to convergent evolution; i.e., they arose independently in these groups. This view was adopted by several other authors, such as Castro and Vari [53] and Mirande [18]. Thus, our results are inconsistent with the current morphology-based topology that identifies the Triportheidae and the Gasteropelecidae as non-sister groups. However, based on morphological analyses, Gregory and Conrad [54] suggested that "*Chalcinus* (=*Triportheus*) is much the nearer to the structural ancestor of *Gasteropelecus*", a hypotheses similar to that found in the present study. In contrast, the putative relationship between *Triportheus* and the Bryconidae, is supported by several authors, including Malabarba [41] and Mirande [18]. However, considering that the support in MP studies was lower than 50% further studies involving more samples and more genes will be necessary for a better discussion about the relationships of these families.

### Phylogenetic position of *Salminus*

*Salminus* is an economically and ecologically important genus, composed of medium to large fishes. Members of the genus are found throughout most of South America, including one trans-Andean representative, *S. affinis*. The taxonomic history of *Salminus* is complex. In the first review of the genus, Eigenmann [55] recognized *S. affinis* (Magdalena River and Upper Amazonas), *S. hilarii* (Paraná, São Francisco, Amazon and Orinoco Rivers), *S. maxillosus* (La Plata basin), and *S. brevidens* (São Francisco River). Presently, four species are recognized: *S. affinis* (trans-Andean species from Magdalena, Rancheria and Sinú Rivers, Colombia), *S. hilarii* (São Francisco, Upper Paraná river basins, Araguaia, Tocantins, Upper Amazonas and Orinoco Rivers), *S. brasiliensis* (La Plata Basin, Jacuí River and Upper Madeira river basin), and *Salminus franciscanus*[56-59]. A morphological distinction among specimens of *S. hilarii* from São Francisco and Upper Paraná river basins and those from Araguaia, Tocantins, Upper Amazonas and Orinoco Rivers was observed by Lima [57] and thus in the present study the first group is here identified as *S. hilarii* and the second as *Salminus sp*.

Morphological studies do not suggest a close relationship between *Salminus* and *Brycon*[4,16,18,57]. Géry [15], without a phylogenetic analyses, included the tribe Salminini in the subfamily Bryconinae. Our study corroborates previous molecular hypothesis that identify *Salminus* as closely related to *Brycon*[1,20,21,51,60]. Moreover, our results show that *Salminus* is a genus interspersed among *Brycon* species, as observed by Calcagnotto *et al.*[20].

Our phylogeny is the first published hypothesis of the evolutionary history of this genus and shows that *Salminus hilarii* is the sister group of all remaining species and that *Salminus sp.* and *S. affinis* and *S. brasiliensis* and *S. franciscanus* are sister species. However, Lima [57] reported similar results in his unpublished thesis.

### Phylogenetic relationships among Bryconidae species

According to our results, the family Bryconidae consists of five main clades. The first clade is composed of four trans-Andean species of *Brycon* and *Chilobrycon. B. chagrensis* is the sister group of all remained species in this clade. After this we have *B. petrosus* in the BEAST analysis and *B. henni* in the ML analysis as a sister group to a monophyletic lineage with two clades, one composed of *B. aff. atrocaudatus* and *B. henni* (*B. petrosus*) and the second consisting of *C. deuterodon*. In their description of the genus *Chilobrycon*, Géry and Rham [61] suggested that this genus belongs to the subfamily Bryconinae and can be differentiated from *Brycon* species primarily by the presence of spatulated and tricuspid teeth and the absence of an upper lip. Although several trans-Andean species of *Brycon* were not available for analysis in the present study, the species *B. chagrensis* was described by Kner (1863) as *Chalcinopsis chagrensis* that was considered a junior synonymous of *B. chagrensis*[4] and thus the whole taxonomy of this group need to be revised.

The second clade is composed of all the species of *Salminus*, as discussed above. The third clade consists of four samples of *Brycon pesu*. Eigenmann [62] proposed that the genus *Holobrycon* include *Brycon pesu* Müller & Troschell, 1841, because adult specimens of this genus lack a fontanel, unlike that observed in all *Brycon* species. Thus, our data reinforce Eigenmann's proposition that *Holobrycon* represents a valid genus; however, these nomenclature changes require close evaluation in future studies.

The fourth clade is comprised of one trans-Andean species of *Brycon* (*B. moorei*) and several *Brycon* species from the Amazon, Orinoco, São Francisco, and Paraná-Paraguay Rivers. The inclusion of *B. moorei* in this clade and the presence of *Brycon* species in our first clade show that the cis- and trans-Andean *Brycon* spp. are not monophyletic. The relationship among cis-Andean species of this clade was not resolved in the present study. Notably, the species *B. orthotaenia, B. orbignyianus* and *B. hilarii* are morphologically very similar (FCT Lima, pers. comm.).

The fifth clade is composed of two lineages: the first contains *B. melanopterus*, *B. falcatus, and B. cf. falcatus* (Amazon and Orinoco) and the second comprises *B. nattereri* (upper Paraná), *B. opalinus, B. insignis, B. vermelha, B. ferox, Brycon sp.*, and *Henochilus* from Brazilian coastal rivers. The sister relationship of *B. melanopterus, B. falcatus*, and *B. cf. falcatus* was expected, as the three species are very similar morphologically. The *Brycon* species from coastal rivers are also morphologically similar (FCT Lima, pers. comm.). The close relationships among those species inhabiting Brazilian coastal regions is interesting as the Eastern Brazilian coastal rivers were connected many times during the Neogene and Quaternary [63], and ancestor groups may have spread throughout this area.

Based on recently collected specimens, Castro *et al.*[51] re-described *Henochilus wheatlandii* and analyzed its relationship with other characiforms using sequences of the genes 12S and 16S. Their results indicated a close relationship between *Brycon* and *Henochilus*, as did those of Hilsdorf *et al.*[60]. *Henochilus* is morphologically very similar to *Brycon* and *Chilobrycon*, with fewer tooth series. This trait may be an autapomorphy of this species [4].

### Origin and diversification of Bryconidae groups

The study of the distribution patterns of freshwater fishes in association with historical biogeography provides an excellent opportunity to test alternative models of evolution of hydrographic basins [64]. Fossil characiforms were described from South America, Africa, Europe, and the Arabian Peninsula [65]. Additionally, a putative characiform fossil was described from Canada [66]. The main lineages of Neotropical freshwater fishes were present in South America by the Lower Cretaceous, and much of their diversification occurred before or during the Paleogene [67-69]. Molecular analyses of Citharinoidei [70] also corroborate the hypotheses of the origin of the order Characiformes as the Lower Cretaceous. Conversely, Eocene-Oligocene articulated specimens of characiforms from the Entre-Córregos Formation, southern Minas Gerais State, eastern Brazil, were described as *Tremembichthys* sp., cf. *Brycon avus*, and an undetermined Characidae [71]. More recently, Weiss *et al.*[72] described two characiforms from this same locality: *Paleotetra entrecorregos* and *P. aiuruoca.* From the Tremembé Formation (Oligocene), five characiform species were described, all based on articulated specimens: *Lignobrycon ligniticus, Megacheirodon unicus, Brycon avus*, *Cyphocharax mosesi,* and *Plesiocurimata alvarengai*[41]. Two other species, *Procharax minor* and *Lignobrycon altus*, were described based on poorly preserved specimens from the Plio-Pleistocene sediments of Nova York shales (northern Brazil) [73].

Using the information available for *Lignobrycon ligniticus,* that met all suggested points signaled by Parham et al. [39] regarding the use of fossils to calibrate molecular trees, we estimate the origin of the Bryconidae at 46.7 ± 11.8 Ma (Figure 4), corresponding to the Eocene-Paleocene. According to López-Fernandes and Alberts [69], major marine regressions exposed large areas of interior floodplains during the Oligocene (as in earlier epochs), allowing dramatic and sometimes rapid expansion of freshwater habitats. The formation of the so-called "foreland basin" [74] may have permitted the rapid expansion of a primitive Bryconidae throughout the proto-Paraná and proto-Amazon system.

Within the Bryconidae, clade 1 originated 37.5 ± 9.7 Ma. This clade is composed of trans-Andean species, including *Chilobrycon deuterodon* and *B. aff. atrocaudatus* from Peru, *B. henni* from Colombia, and *Brycon chagrensis* and *B. petrosus* from Panama. The last closure of the Panama Isthmus is generally considered to have occurred between 3.1-2.8 Ma [75,76]. However, some alternative hypotheses regarding faunal exchanges between Central and South America have been proposed. According to Haq *et al.*[77], during the lower middle Miocene, sea levels were generally very high, but two sea-level drops of almost 100 m may have occurred between 17 and 15 Ma, which could have permitted the migration of fish from South to Central America. Recent geological studies [78,79] suggest that the Panamanian land bridge may be much older (ca. 23-25 Ma). The GAARlandia hypothesis [80] proposes that a geological connection was present between the central part of Central America and South America 32 Ma, formed via a land bridge between the Greater Antilles and the Aves Islands Ridge. Finally, a Cretaceous Island Arc [81] has been proposed linking Central America, the Greater Antilles, and South America 80–70 Ma. Some have argued that this Cretaceous Island Arc may have persisted until 49 Ma [82].

Although some previous molecular studies suggest that some primary freshwater fishes from Central America originated after the formation of the Panama landbridge at the end of Tertiary [83,84], an earlier colonization of Mesoamerica has been proposed for other freshwater fishes [85-90]. Additionally, molecular studies of terrestrial taxa support a late Oligocene/early Miocene land connection between Central and South America [91-96]. Thus, although our molecular clock may be limited by the absence of older Bryconidae fossils and, potentially, additional samples, the results obtained are in accordance with an ancient invasion of Central America, dating at least from the origin of the ancestor of *B. chagrensis*, 20.3 ± 5.0 Ma and could be related with the changes in sea level during the lower middle Miocene [77]. However, the hypothesis of multiple invasions, as proposed by Reeves and Bermingham [84], is also in accordance with our data since the ancestor of *B. petrosus* originated at 10.1 ± 2.8 Ma (Figure 4).

Clade 2, comprised exclusively of *Salminus*, originated 29.6 ± 7.8 Ma (Figure 4). A recent *Salminus* fossil found in eastern Argentina was dated as from the early late Miocene (Tortonian - 7.1 to 11.2 Ma) [97], consistent with our hypotheses. The first cladogenesis in this group gave origin to the ancestor of *S. hilarii* and the ancestor of the remaining lineages. *S. hilarii* has a restricted distribution in the Upper Paraná and São Francisco Rivers [57]. Our data suggest that the split between lineages of *S. brasiliensis* (in the Paraná, Paraguay and Uruguay River basins and adjacent areas) and *S. franciscanus* (São Francisco River basin) occurred at 5.9 ± 1.8 Ma (Figure 4). In his study of *Hypostomus*, Montoya-Burgos [98] identified a separation time of his samples from the Paraná and São Francisco Rivers (Clade D3) of between 5.7 and 6.4 Ma, a result similar to that of the present study and that of Beurlen (1970; cited by [99]), who suggested that a connection between these basins may have been present from the Tertiary until 1.8 Ma. The cladogenesis that gave origin to *Salminus sp.* (Amazon basin) and *S. affinis* (Magdalena River basin) occurred at 3.6 ± 1.5 Ma (Figure 4). According to Hoorn *et al.*[99], the most intense periods of formation of the northern Andean mountains occurred from the late middle Miocene (~12 Ma) to early Pliocene (~4.5 Ma), a timeframe close to the putative separation time we identified between *Salminus sp.* and *S. affinis*.

Clade 3, consisting of *Brycon pesu*, originated 26.7 ± 6.4 Ma. Although this clade is represented by a single species in our study, recent analysis have shown that it is a species complex, composed by several underscribed species (FCT Lima, pers. comm.). This clade is widely distributed throughout the Amazon and Orinoco River basins, as well as in rivers from Guyana, Suriname and French Guiana [59], but our specimens are only from the Amazon basin. Our data suggest a rapid diversification within the last 6.7 ± 2.0 Ma. This period approximately coincides with the final formation of the Amazon and Orinoco drainages [99] and other coastal drainages in north South America. Additional analysis with samples from other drainages and the possible new species will be necessary to better understand the diversification of this group.

Clades 4 and 5 originated 22.3 ± 6.0 Ma. Clade 4 has two main lineages. *Brycon moorei* (Magdalena River basin) ancestor diverged from the other species at 18.2 ± 4.8 Ma. This old separation time coincides with the first peak of mountain building in the Northern Andes (late Oligocene to early Miocene, approximately 23 Ma), coinciding with the diversification of the first modern mountain plant and animal genera [99]. Although in our general phylogeny (Figure 3) the remained species in the clade 4 were not statistically well separated in all analyses the Bayesian inference suggest that several lineages originated in different times. Thus, *B. orthotaenia* (São Francisco River basin) ancestor diverged from the other species of this group at 15.2 ± 4.0 Ma (Figure 4). Several authors have suggested that the fish fauna of the São Francisco basin are a hybrid combination of groups from adjacent basins [100,101]. Thus, the present data suggest that those connections may have been present since the early Miocene. *B. amazonicus* (Amazon, Orinoco and Essequibo River basins) ancestor diverged from *B. hilarii* (Paraguay River basin) 12.6 ± 3.3 Ma (Figure 4). According to Lundberg *et al.*[67], the headwater capture of the Upper Paraguay by the Amazonas occurred due to the shift south to the Michicola Arch, 11.8 - 10.0 Ma, a time period very close to that we found. The divergence of *B. orbygnianus* (La Plata River basin) ancestor from *B. gouldingi* ancestor (Tocantins River basin) 10.7 ± 3.1 Ma may also have occurred after the shift of the Michicola Arch.

In clade 5, *Brycon falcatus* (Amazon and Orinoco River basins and rivers in Guyana, Suriname and French Guiana), *B. cf. falcatus* (Culuene River, a tributary of Xingu River) and *B. melanopterus* (Amazon River basin) ancestors diverged approximately 18.4 ± 5.2 Ma from the remaining species of this clade that inhabit the Paraná-Paraguay, São Francisco and coastal rivers in eastern Brazil (Figure 4). This time coincides with the formation of Chapare Buttress, a structural divide that formed between the paleo-Amazonas-Orinoco and Paraguay basins 30 - 20 Ma [67].

The divergence of *B. falcatus* ancestor from the Orinoco River (sample number 15563) from *B. falcatus* ancestor from the Negro River (sample number 32395) occurred 4.4 ± 1.4 Ma (Figure 4). Studies in the Callichthyinae have shown that specimens of *Hoplosternum litoralle* and *Megalechis picta* from the Amazon and Orinoco diverged approximately 11.7 to 6 Ma, respectively [102]. These data are in accordance with the separation of the Amazon and Orinoco with the formation of Vaupes Arch (late Miocene) and subsequent changes up until the Holocene, when the Andean mountains attained their present configuration [103].

The remaining species in clade 5 are restricted to the Upper Paraná and São Francisco Rivers (*Brycon nattereri*) and coastal rivers (*B. opalinus*, *Henochilus wheatlandii*, *B. vermelha*, *B. insignis*, *B. ferox* and *Brycon sp.*) and originated 15.1 ± 3.9 Ma (Figure 4). In this group, *B. opalinus* from the Paraiba do Sul River is the sister group of the remaining species. This species occurs in the area of the Tremembé formation (Oligocene), where the fossils *Brycon avus* and *Lignobrycon ligniticus* were found [41]. Considering *B. avus* as an Oligocene species it is more possible that it is the sister group of clades 3 to 5 than close relative to the *Brycon* species that today inhabit the Brazilian coastal area. A future phylogenetic analyses including this species will be necessary to test this hypotheses.

The relationship between the *Brycon* species of the Amazon and south coastal rivers (clade 5) may be due to the dispersal of a now extinct form from the Paraguay River that dispersed from the south northward into Paraguay and the south coastal rivers as could be *B. avus*. The other species in this group diverged more recently (end of the Miocene and Pliocene), and events such as global climate oscillations and eustatic sea-level fluxes [63] may have produced ancient connections among these coastal rivers, allowing species dispersion and speciation.

In summary, our results align with several geological events in South America, but suggest an old colonization of Central America. However, further studies that include several as yet unsampled *Brycon* species are necessary to better understand the relationships among some lesser known species such as *B. coquenani, B. polylepis* and *B. whitei*, which could provide new insights into the relationships among *Brycon* species as well as the origin of some taxa.

## Conclusion

Bryconidae is composed by five main clades, including the genera *Brycon*, *Chilobrycon*, *Henochilus* and *Salminus*, but a taxonomic review of these groups is needed. Our results points to a possible ancient invasion of Central America, dating about 20.3 ± 5.0 Ma (late Oligocene/early Miocene), to explain the occurrence of *Brycon* in Central America.

## Competing interests

The authors declare that they have no competing interests.

## Authors’ contribution

KTA and CO participated equally in the design of the study. KTA and TCM did the most laboratory experiments. KTA and CO analyzed parts of the data and did phylogenetic analyses. All authors discussed results. KTA and CO wrote substantial parts of the manuscript. All authors read and approved the final manuscript.

## Supplementary Material

Additional file 1 Species analyzed, collection number, specimen number, and GenBank accession numbers. Click here for file

Additional file 2 Sequences of primers used in present study. Click here for file
