# Supplementary material for: Systematic and historical biogeography of the Bryconidae (Ostariophysi: Characiformes) suggesting a new rearrangement of its genera and an old origin of Mesoamerican ichthyofauna
Source: BMC Evol Biol. 2014 Jul 8;14:152. doi: 10.1186/1471-2148-14-152 (PMC4109779; doi:10.1186/1471-2148-14-152)
Supplement: Additional file 1 — Species analyzed, collection number, specimen number, and GenBank accession numbers. [file 1471-2148-14-152-S1.pdf]

Additional File 1: **Details of the specimens used in the phylogenetic analysis**.

Group/species	Voucher	Specimen	16S	CytB	Myh6	Rag1	Rag2
**Acestrorhynchidae**							
Acestrorhynchus cf. nasutus[1]	LBP 7035	34110	HQ171379	HQ289666	HQ289087	HQ289280	-
Acestrorhynchus falcatus[1]	LBP 4191	23707	HQ171312	HQ289601	HQ289022	HQ289214	HQ289408
Acestrorhynchus lacustris[1]	LBP 2158	15173	HQ171250	HQ289541	HQ288960	HQ289157	HQ289348
Acestrorhynchus pantaneiro[1]	LBP 3755	22014	HQ171288	HQ289577	HQ288998	HQ289192	HQ289385
Gilbertolus maracaiboensis[1]	LBP 6107	29552	HQ171362	HQ289649	HQ289070	HQ289263	HQ289456
Gnathocharax steindachneri[1]	LBP 4496	24494	HQ171325	-	HQ289034	-	HQ289421
Heterocharax macrolepis[1]	LBP 4494	24485	HQ171323	HQ289612	HQ289032	HQ289225	HQ289419
Hoplocharax goethei[1]	LBP 4495	24489	HQ171324	HQ289613	-	HQ289226	-
Roestes ogilviei[1]	LBP 8157	38066	HQ171410	HQ289697	HQ289118	HQ289311	HQ289503
**Alestidae**							
Alestes sp.[1]	LBP 7530	35376	HQ171390	HQ289677	HQ289098	HQ289291	HQ289483
Alestopetersius caudalis[3]			AY788019	AY791401	-	-	AY804078
Bathyaethiops breuseghemi[3]	AMNH 233422		AY788068	AY791430	-	-	AY804113
Brycinus carolinae[3]	AMNH 233628		AY787960	AY791357	-	-	-
Brycinus longipinnis	LBP 7529	35375	HQ171389	HQ289676	HQ289097	HQ289290	HQ289482
Bryconaethiops sp. [3]			AY787983	AY791374	-	-	AY804047
Hydrocynus brevis[3]	AMNH 22644		AY788018	AY791400	-	-	AY804077
Ladigesia roloffi[3]	AMNH 233394		AY788046	AY791417	-	-	AY804097
Micralestes sp.	LBP 2342	15946	HQ171258	HQ289549	HQ288968	HQ289165	HQ289356
Phenacogrammus interruptus	LBP 2637	17293	HQ171263	HQ289554	HQ288973	HQ289170	HQ289361
**Anostomidae**							
Anostomus ternetzi[1]	LBP 4375	24146	HQ171317	HQ289606	HQ289026	HQ289219	HQ289413
Leporinus fasciatus[1]	LBP 4459	24381	HQ171321	HQ289610	HQ289030	HQ289223	HQ289417
Schizodon fasciatus[1]	LBP 3046	19130	HQ171270	HQ289559	HQ288980	HQ289177	HQ289367
Schizodon fasciatus[1]	LBP 3994	23098	HQ171308	HQ289597	HQ289018	HQ289210	HQ289404
**Bryconidae**							
Brycon amazonicus[1]	LBP 2187	15565	HQ171251	HQ289542	HQ288961	HQ289158	HQ289349
		15567	KF779969	KF780010	KF780046	KF780085	KF780117
Brycon amazonicus	LBP 2859	18988	KF779970	KF780011	KF780047	KF780086	KF780118
Brycon amazonicus	LBP 834	8835	KF779968	KF780009	-	KF780084	KF780116
Brycon aff. *atrocaudatus*	LBP 1356	17096	KF779971	KF780012	-	-	KF780119
Brycon chagrensis	LBP 2749	18510	KF779972	KF780013	KF780048	KF780087	KF780120
Brycon falcatus	LBP 2668	15563	KF779973	KF780014	KF780049	KF780088	KF780121
Brycon falcatus	LBP 5146	26278	KF779974	KF780015	KF780050	KF780089	KF780122
Brycon falcatus	LBP 6878	32395	KF779975	KF780016	KF780051	KF780090	KF780123
Brycon cf. falcatus	LBP 8109	37580	KF779976	KF780017	KF780052	KF780091	KF780124
		37581	KF779977	KF780018	KF780053	KF780092	KF780125
Brycon ferox	LBP 2855	18979	KF779978	KF780019	KF780054	KF780093	KF780126
Brycon ferox	LBP 8099	37528	KF779979	KF780020	KF780055	KF780094	KF780127
Brycon ferox	LBP 8100	37529	KF779980	KF780021	KF780056	KF780095	KF780128
Brycon gouldingi	LBP 3130	19203	KF779981	KF780022	KF780057	KF780096	KF780129
Brycon henni	LBP 2857	18984	KF779982	-	KF780058	KF780097	KF780130
Brycon hilarii	LBP 3805	21895	KF779983	KF780023	KF780059	KF780098	KF780131
Brycon hilarii	LBP 2766	17634	KF779984	KF780024	KF780060	KF780099	KF780132
Brycon hilarii	LBP 4676	24810	KF779985	KF780025	KF780061	KF780100	KF780133
Brycon insignis[1]	LBP 2369	16075	HQ171260	HQ289551	HQ288970	HQ289167	HQ289358
Brycon melanopterus	LBP 9778	38906	KF779986	KF780026	KF780062	KF780101	KF780134
		38907	KF779987	KF780027	KF780063	KF780102	KF780135
Brycon moorei	LBP 2858	18986	KF779988	KF780028	KF780064	-	KF780136
Brycon moorei	LBP 12817	55010	KF779989	KF780029	-	-	KF780137
Brycon nattereri	LBP 2856	18981	KF779990	KF780030	KF780065	-	KF780138
		18982	KF779991	KF780031	KF780066	-	KF780139
Brycon nattereri	LBP 8101	37541	KF779992	KF780032	KF780067	KF780103	KF780140
Brycon orbignyanus	LBP 2746	18004	KF779993	KF780033	KF780068	KF780104	KF780141
Brycon opalinus	LBP 6303	29001	KF779994	KF780034	KF780069	KF780105	KF780142
Brycon opalinus	LBP 6306	29349	KF779995	KF780035	KF780070	KF780106	KF780143
Brycon orthotaenia	LBP 249	4215	KF779996	KF780036	KF780071	KF780107	KF780144
Brycon pesu	LBP 8111	37578	KF779997	KF780037	KF780072	-	KF780145
		37579	KF779998	KF780038	KF780073	-	KF780146
Brycon pesu	LBP 5320	26930	KF779999	KF780039	KF780074	-	KF780147
Brycon pesu	LBP 9409	42567	KF780000	KF780040	KF780075	KF780108	KF780148
Brycon petrosus	LBP 2750	18504	KF780001	KF780041	KF780076	KF780109	KF780149
Brycon vermelha	LBP 9066	42508	KF780002	-	KF780077	KF780110	-
Brycon sp.	LBP 5837	28350	KF780008	KF480045	KF780083	KF780115	KF780155
Chilobrycon deuterodon	LBP 9334	45001	KF780003	KF780042	KF780078	-	KF780150

		45002	KF780004	KF480043	KF780079	KF780111	KF780151
Henochilus wheatlandii[1]	LBP 1221	25846	HQ171335	HQ289624	HQ289044	HQ289237	HQ289431
Salminus affinis	LBP 12817	55009	KF780005	-	KF780080	KF780112	KF780152
Salminus brasiliensis[1]	LBP 850	9025	HQ171437	HQ289722	HQ289145	HQ289336	HQ289528
Salminus franciscanus[1]	LBP 8090	37503	HQ171401	HQ289688	HQ289109	HQ289302	HQ289494
Salminus hilarii	LBP 84	7615	KF780006	-	KF780081	KF780113	KF780153
Salminus iquitensis	LBP 8160	38065	KF780007	KF480044	KF780082	KF780114	KF780154
**Chalceidae**							
Chalceus epakros[1]	LBP 5443	26504	HQ171341	HQ289630	-	-	HQ289436
Chalceus erythrurus[1]	LBP 4211	22727	HQ171297	HQ289586	HQ289007	HQ289201	HQ289394
**Characidae/Stevardiinae**							
Bryconadenos tanaothoros[2]	MCP 40399		FJ748980	-	-	-	FJ749089
Bryconamericus emperador[1]	LBP 2754	18528	HQ171266	HQ289557	HQ288976	HQ289173	HQ289363
Bryconamericus exodon[1]	LBP 7123	34200	HQ171380	HQ289667	HQ289088	HQ289281	HQ289474
Ceratobranchia cf. delotaenia[1]	LBP 3257	20042	HQ171278	HQ289567	HQ288988	HQ289183	HQ289375
Corynopoma riisei[2]			FJ749009	-	-	-	FJ749095
Creagrutus peruanus[1]	LBP 3267	20057	HQ171279	HQ289568	HQ288989	HQ289184	HQ289376
Cyanocharax alburnus[1]	LBP 4746	25516	HQ171333	HQ289622	HQ289042	HQ289235	HQ289429
Gephyrocharax atracaudatus[1]	LBP 2753	18519	HQ171265	HQ289556	HQ288975	HQ289172	HQ289362
Glandulocauda melanogenys[1]	LBP 4507	24538	HQ171320	HQ289609	HQ289029	HQ289222	HQ289416
Hemibrycon taeniurus[1]	LBP 6847	33168	HQ171369	HQ289656	HQ289077	HQ289270	HQ289463
Hypobrycon maromba[1]	LBP 6750	33174	HQ171375	HQ289662	HQ289083	HQ289276	HQ289469
Knodus meridae[1]	LBP 7569	15818	HQ171257	HQ289548	HQ288967	HQ289164	HQ289355
Lophiobrycon weitzmani[1]	LBP 1225	38090	HQ171411	HQ289698	HQ289119	HQ289312	HQ289504
Markiana nigripinnis[1]	LBP 663	8038	HQ171432	-	HQ289140	HQ289331	HQ289524
Mimagoniates inequalis[1]	LBP 3383	21274	HQ171282	HQ289571	HQ288992	HQ289186	HQ289379
Mimagoniates microlepis[1]	LBP 1225	11077	HQ171240	HQ289531	HQ288950	HQ289147	HQ289338
Odontostoechus lethostigmus[1]	LBP 6752	33171	HQ171372	HQ289659	HQ289080	HQ289273	HQ289466
Piabarchus analis[1]	LBP 8514	38382	HQ171412	HQ289699	HQ289120	HQ289313	HQ289505
Piabina argêntea[1]	LBP 3509	21306	HQ171283	HQ289572	HQ288993	HQ289187	HQ289380
Planaltina britskii	LBP 2598	17243	HQ171262	HQ289553	HQ288972	HQ289169	-
Pseudocorynopoma heterandria[1]	LBP 2862	18570	HQ171268	-	HQ288978	HQ289175	HQ289365
Tyttocharax madeirae[1]	LBP 5145	33166	HQ171368	HQ289655	HQ289076	HQ289269	HQ289462
Xenurobrycon pteropus[1]	LBP 9054	42218	HQ171423	HQ289709	HQ289131	HQ289324	HQ289516
**Characidae/Clade B**							
**Aphyocharacinae**							
Aphyocharacidium bolivianum[1]	LBP 9055	42219	HQ171424	HQ289710	HQ289132	HQ289325	HQ289517
Aphyocharax alburnus[1]	LBP 1587	11774	HQ171242	HQ289533	HQ288952	HQ289149	-
Aphyocharax anisitsi[1]	LBP 3764	22190	HQ171292	HQ289581	HQ289002	HQ289196	HQ289389
Aphyocharax pusillus[1]	LBP 4046	22920	HQ171301	HQ289590	HQ289011	HQ289203	HQ289397
Leptagoniates steindachneri[1]	LBP 4137	23661	HQ171311	HQ289600	HQ289021	HQ289213	HQ289407
Paragoniates alburnus[1]	LBP 9208	43156	HQ171426	HQ289712	HQ289134	HQ289326	HQ289519
Phenagoniates macrolepis[1]	LBP 6105	35623	HQ171391	HQ289678	HQ289099	HQ289292	HQ289484
Prionobrama paraguayensis[1]	LBP 3230	19465	HQ171272	HQ289261	HQ288982	-	HQ289369
Prionobrama paraguayensis[1]	LBP 3230	19468	HQ171273	HQ289262	HQ288983	HQ289179	HQ289370
Xenagoniates bondi[1]	LBP 3074	19694	HQ171274	HQ289563	HQ288984	-	HQ289371
**Characinae**							
Acestrocephalus sardina[1]	LBP 6876	33172	HQ171373	HQ289660	-	HQ289274	HQ289467
Charax leticiae[1]	LBP 1480	12700	HQ171244	HQ289535	HQ288954	HQ289151	HQ289342
Cynopotamus kincaidi[1]	LBP 3225	19449	HQ171271	HQ289560	HQ288981	HQ289178	HQ289368
Cynopotamus venezuelae[1]	LBP 6132	29515	HQ171359	HQ289648	HQ289068	HQ289261	HQ289454
Galeocharax knerii[1]	LBP 3496	20164	HQ171280	HQ289569	HQ288990	-	HQ289377
Phenacogaster calverti[1]	LBP 5582	27299	HQ171347	HQ289636	HQ289056	HQ289249	HQ289442
Roeboides guatemalensis[1]	LBP 2755	18529	HQ171267	-	-	-	HQ289364
**Cheirodontinae**							
Aphyocheirodon hemigrammus[1]	LBP 8306	40025	HQ171413	HQ289700	HQ289121	HQ289314	-
Cheirodon ibicuhiensis[1]	LBP 4777	25598	HQ171334	HQ289623	HQ289043	HQ289236	HQ289430
Cheirodon killiani[1]	LBP 3115	19803	HQ171275	HQ289564	-	-	HQ289372
Compsura heterura[1]	LBP 4733	24984	HQ171332	HQ289621	HQ289041	HQ289234	HQ289428
Ctenocheirodon pristis [1]	LBP 5699	27603	HQ171350	HQ289639	HQ289059	HQ289252	HQ289445
Heterocheirodon yatai[1]	LBP 4872	24954	HQ171330	HQ289619	HQ289039	HQ289232	HQ289426
Kolpotocheirodon theloura[1]	LBP 5033	25982	HQ171336	HQ289625	HQ289045	HQ289238	HQ289432
Macropsobrycon uruguayanae[1]	LBP 6039	29061	HQ171355	HQ289644	HQ289064	HQ289257	HQ289450
Nanocheirodon insignis[1]	LBP 6104	27476	HQ171349	HQ289638	HQ289058	HQ289251	HQ289444
Odontostilbe fugitiva[1]	LBP 4052	22932	HQ171302	HQ289591	HQ289012	HQ289204	HQ289398
Odontostilbe sp.1 [1]	LBP 4650	22626	HQ171296	HQ289585	HQ289006	HQ289200	HQ289393
Prodontocharax melanotus[1]	AMNH	233264	HQ 171239	HQ289530	HQ288949	HQ289146	HQ289337
Pseudocheirodon arnoldi[1]	STRI	5	HQ171430	HQ289715	HQ289138	HQ289329	HQ289522
Saccoderma melanostigma[1]	LBP 6103	27475	HQ171348	HQ289637	HQ289057	HQ289250	HQ289443
Serrapinnus calliurus[1]	LBP 3731	22121	HQ171291	HQ289580	HQ289001	HQ289195	HQ289388
Serrapinnus heterodon[1]	LBP 9039	37551	HQ171403	HQ289690	HQ289111	HQ289304	HQ289496

Serrapinnus piaba[1]	LBP 8972	41813	HQ171416	HQ289703	HQ289124	HQ289317	HQ289509
Spintherobolus ankoseion[1]	LBP 4725	24957	HQ171331	HQ289620	HQ289040	HQ289294	HQ289427
Spintherobolus broccae[1]	LBP 3916	22558	HQ171294	HQ289583	HQ289004	HQ289233	HQ289391
Spintherobolus leptoura[1]	LBP 7544	36098	HQ171393	HQ289680	HQ289101	HQ289198	HQ289486
**Tetragonopterinae**							
Tetragonopterus argenteus[1]	LBP 3758	22029	HQ171289	HQ289578	HQ288999	HQ289193	HQ289386
Tetragonopterus chalceus[1]	LBP 8268	37556	HQ171405	HQ289692	HQ289113	HQ289306	HQ289498
**Clade B *Incertae sedis***							
Microschemobrycon casiquiare[1]	LBP 8161	38058	HQ171409	HQ289696	HQ289117	HQ289310	HQ289502
Exodon paradoxus[1]	LBP 4006	23040	HQ171306	HQ289595	HQ289016	HQ289208	HQ289402
Roeboexodon guyanensis[1]	LBP 5315	26921	HQ171345	HQ289634	HQ289054	HQ289247	HQ289440
**Characidae/Clade C**							
**Rhoadsiinae**							
Carlana eigenmanni[1]	LBP 3300	19864	HQ171276	HQ289565	HQ288986	HQ289181	-
Carlana eigenmanni[1]	LBP 3301	19865	HQ171277	HQ289566	HQ288987	HQ289182	-
**Stethaprioninae**							
Brachychalcinus copei[1]	LBP 192	8853	HQ171435	HQ289720	HQ289143	HQ289334	HQ289527
Orthospinus franciscensis[1]	LBP 8105	37555	HQ171404	-	HQ289112	HQ289305	HQ289497
Poptella paraguayensis[1]	LBP 3732	21986	HQ171286	HQ289575	HQ288996	HQ289190	-
Stethaprion crenatum[1]	LBP 4078	22994	HQ171305	HQ289594	HQ289015	HQ289207	HQ289401
**Clade C *Incertae sedis***							
Aphyodite grammica[1]	LBP 9050	42214	HQ171421	HQ289707	HQ289129	HQ289322	HQ289514
Astyanacinus moorii[1]	LBP 5783	28195	HQ171352	HQ289641	HQ289061	HQ289254	HQ289447
Astyanax aeneus[1]	LBP 8938	42019	HQ171418	-	HQ289126	HQ289319	HQ289511
Astyanax jordani[1]	LBP 4527	24599	HQ171327	HQ289616	HQ289036	HQ289229	HQ289423
Astyanax mexicanus[1]	LBP 8937	42016	HQ171417	-	HQ289125	HQ289318	HQ289510
Bario steindachneri[1]	LBP 4389	24187	HQ171319	HQ289608	HQ289028	HQ289221	HQ289415
Bramocharax baileyi[1]	LBP 8940	42025	HQ171420	HQ289706	HQ289128	HQ289321	HQ289513
Bramocharax caballeroi[1]	LBP 8939	42022	HQ171419	HQ289705	HQ289127	HQ289320	HQ289512
Bryconella pallidifrons[1]	LBP 4646	24696	HQ171329	HQ289618	HQ289038	HQ289231	HQ289425
Coptobrycon bilineatus[1]	LBP 3809	33169	HQ171370	HQ289657	HQ289078	HQ289271	HQ289464
Ctenobrycon hauxwellianus[1]	LBP 4095	23538	HQ171310	HQ289599	HQ289020	HQ289212	HQ289406
Deuterodon iguape[1]	LBP 6827	33065	HQ171366	HQ289653	HQ289074	HQ289267	HQ289460
Gymnocorymbus ternetzi[1]	LBP 3737	21989	HQ171287	HQ289576	HQ288997	HQ289191	HQ289384
Hasemania sp. [1]	LBP5967	28455	HQ171354	HQ289643	HQ289063	HQ289256	HQ289449
Hemigrammus marginatus[1]	LBP 6292	29419	HQ171357	HQ289646	HQ289066	HQ289259	HQ289452
Hemigrammus ulreyi[1]	LBP 7604	36267	HQ171394	HQ289681	HQ289102	HQ289295	HQ289470
Hollandichthys multifasciatus[1]	LBP 698	8791	HQ171434	-	HQ289142	HQ289333	HQ289526
Hyphessobrycon eques[1]	LBP 7615	36278	HQ171395	HQ289682	HQ289103	HQ289296	HQ289488
Hyphessobrycon megalopterus[1]	LBP 7613	36932	HQ171397	HQ289684	HQ289105	HQ289298	HQ289490
Hyphessobrycon reticulatus[1]	LBP 1049	8939	HQ171436	HQ289721	HQ289144	HQ289335	HQ289487
Inpaichthys kerri[1]	LBP 4526	24597	HQ171326	HQ289615	HQ289035	HQ289228	HQ289422
Jupiaba anteroides[1]	LBP 7067	34380	HQ171381	HQ289668	HQ289089	HQ289282	HQ289475
Jupiaba cf. acanthogaster[1]	LBP 7935	37269	HQ171399	HQ289686	HQ289107	HQ289300	HQ289492
Moenkhausia xinguensis[1]	LBP 6101	28443	HQ171353	HQ289642	HQ289062	HQ289255	HQ289448
Myxiops aphos[1]	LBP 7184	33173	HQ171374	HQ289661	HQ289082	HQ289275	-
Nematobrycon palmeri[1]	LBP 6124	33165	HQ171367	HQ289654	HQ289075	HQ289268	HQ289461
Nematocharax venustus[1]	LBP 8106	37557	HQ171406	HQ289693	HQ289114	HQ289307	HQ289499
Oligosarcus paranensis[1]	LBP 3926	22582	HQ171295	HQ289584	HQ289005	HQ289199	HQ289392
Oligosarcus hepsetus[1]	LBP 2377	16055	HQ171259	HQ289550	-	-	HQ289357
Paracheirodon axelrodi[1]	LBP 4472	24425	HQ171322	HQ289611	HQ289031	HQ289224	HQ289418
Parecbasis cyclolepis[1]	LBP 9053	42217	HQ171422	HQ289708	HQ289130	HQ289323	HQ289515
Pristella maxillaris[1]	LBP 2221	15637	HQ171255	HQ289546	HQ288965	HQ289162	HQ289353
Probolodus heterostomus[1]	LBP 6454	29141	HQ171356	HQ289645	HQ289065	HQ289258	HQ289451
Psellogrammus kennedyi[1]	LBP 6578	31813	HQ171365	HQ289652	HQ289073	HQ289266	HQ289459
Rachoviscus crassiceps[1]	LBP 7146	33170	HQ171371	HQ289658	HQ289079	HQ289272	HQ289465
Stygichthys typhlops[1]	LBP 8107	37558	HQ171407	HQ289694	HQ289115	HQ289308	HQ289500
Thayeria obliqua[1]	LBP 5743	26891	HQ171344	HQ289633	HQ289053	HQ289246	HQ289439
Characidae Gen. & sp. nov. [1]	LBP 7243	33196	HQ171376	HQ289663	-	-	HQ289470
**Chilodontidae**							
Chilodus punctatus[1]	LBP 4090	23527	HQ171309	HQ289598	-	-	-
Caenotropus labyrinthicus[1]	LBP 1828	12912	HQ171247	HQ289538	-	-	HQ289345
Caenotropus labyrinthicus[1]	LBP 9216	43161	HQ171428	-	HQ289136	HQ289327	-
**Citharinidae**							
Citharinus sp. [1]	LBP 7528	35374	HQ171388	HQ289675	-	-	HQ289481
**Crenuchidae**							
Characidium laterale[1]	LBP 7614	36938	HQ171398	HQ289685	HQ289106	HQ289299	HQ289491
Characidium pterostictum[1]	LBP 2132	21388	HQ171284	HQ289573	HQ288994	HQ289188	HQ289381

Crenuchus spilurus[1]	LBP 6907	33264	HQ171377	HQ289664	HQ289085	HQ289278	HQ289471
Melanocharacidium sp.[3]	AMNH 233321		AY788083	AY791439	-	-	AY804126
Poecilocharax weitzmani[1]	LBP 7078	40500	HQ171414	HQ289701	HQ289122	HQ289315	HQ289507
**Ctenoluciidae**							
Boulengerella lateristriga[1]	LBP 7094	34623	HQ171382	HQ289669	-	-	-
Boulengerella maculata[1]	LBP 3996	23092	HQ171307	HQ289596	-	-	-
Boulengerella maculata[1]	LBP 4241	22733	HQ171298	HQ289587	-	-	-
Ctenolucius hujeta[1]	LBP 6131	29532	HQ171360	-	-	-	-
Ctenolucius hujeta[1]	LBP 6131	29533	HQ171361	-	-	-	-
**Curimatidae**							
Curimatella dorsalis[1]	LBP3759	22034	HQ171290	HQ289579	HQ289000	HQ289194	HQ289387
Cyphocharax gouldingi[1]	LBP 1537	11889	HQ171243	HQ289534	HQ288953	HQ289150	HQ289342
Cyphocharax magdalenae[1]	LBP6109	29560	HQ171363	HQ289650	HQ289071	HQ289264	-
Potamorhina altamazonica[1]	LBP2571	17020	HQ171261	HQ289552	HQ288971	HQ289168	HQ289359
Steindachnerina insculpta[1]	LBP5185	26336	HQ171339	HQ289628	HQ289048	HQ289241	HQ289435
**Cynodontidae**							
Cynodon gibbus[1]	LBP 1619	11672	HQ171241	HQ289532	HQ288951	HQ289148	HQ289339
Hydrolycus scomberoides[1]	LBP 3031	19115	HQ171269	HQ289558	HQ288979	HQ289176	HQ289366
Rhaphiodon vulpinus[1]	LBP 4064	22942	HQ171303	HQ289592	HQ289013	HQ289205	-
**Distichodontidae**							
Distichodus sp. [1]	LBP 7526	35371	HQ171385	HQ289672	HQ289093	HQ289286	HQ289478
Distichodus sp. [1]	LBP 7526	35372	HQ171386	HQ289673	HQ289094	HQ289287	HQ289479
Hemigrammocharax multifasciatus[3]	RUSI63497		AY788029	AY791407	-	-	AY804085
Ichthyborus sp. [3]	AMNH 233626		AY788038	AY791412	-	-	AY804092
Neolebias trilineatus[3]	AMNH 233439		AY788063	AY791425	-	-	AY804108
Xenocharax spilurus[3]	AMNH 231548		AY788085	AY791441	-	-	-
**Erythrinidae**							
Erythrinus erythrinus[1]	LBP 5212	26378	HQ171340	HQ289629	HQ289049	HQ289242	-
Hoplerythrinus unitaeniatus[1]	LBP 8025	37723	HQ171408	-	HQ289116	HQ289309	-
Hoplias aimara[1]	LBP 7837	36847	HQ171396	HQ289683	HQ289104	HQ289248	-
Hoplias malabaricus[1]	LBP 5539	27219	HQ171346	HQ289635	HQ289055	HQ289297	-
Hoplias microlepis[1]	LBP 2763	18503	HQ171264	HQ289555	HQ288974	HQ289171	-
**Gasteropelecidae**							
Carnegiella marthae[1]	LBP 4199	23793	HQ171313	HQ289602	HQ289023	HQ289216	HQ289409
Carnegiella strigata[1]	LBP 4200	23798	HQ171314	HQ289603	-	HQ289215	HQ289410
Gasteropelecus sternicla[1]	LBP 4070	22975	HQ171304	-	HQ289014	HQ289206	HQ289400
Thoracocharax stellatus[1]	LBP 7534	35343	HQ171384	HQ289671	HQ289092	HQ289285	-
**Iguanodectidae**							
Bryconops affinis[1]	LBP 262	4168	HQ171415	HQ289702	HQ289123	HQ289316	HQ289508
Iguanodectes geisleri[1]	LBP 4266	23840	HQ171316	HQ289605	-	-	HQ289412
Piabucus melanostomus[1]	LBP 5109	26150	HQ171338	HQ289627	HQ289047	-	-
**Hemiodontidae**							
Anodus orinocensis	LBP 2210	15614	HQ171254	HQ289545	HQ288964	HQ289161	HQ289352
Argonectes robertsi	LBP 1804	13167	HQ171249	HQ289540	HQ288959	HQ289156	HQ289347
Bivibranchia velox	LBP 5757	28123	HQ171351	HQ289640	-	-	HQ289446
Hemiodus immaculatus	LBP1725	12849	HQ171246	HQ289537	HQ288956	HQ289153	HQ289344
**Hepsetidae**							
Hepsetus odoe	LBP 7527	35373	HQ171387	HQ289674	HQ289095	HQ289288	HQ289480
**Lebiasinidae**							
Copella nattereri	LBP 4377	24148	HQ171431	HQ289607	HQ289027	HQ289220	HQ289414
Copella nattereri	LBP 536	7140	HQ171318	HQ289716	HQ289139	HQ289330	HQ289523
Pyrrhulina australis	LBP 3784	22333	HQ171293	-	-	HQ289197	HQ289390
Pyrrhulina cf. zigzag	LBP 8005	37473	HQ171400	HQ289687	HQ289108	HQ289301	HQ289493
**Parodontidae**							
Apareiodon affinis[1]	LBP 4591	24665	HQ171328	HQ289617	HQ289037	HQ289230	HQ289424
Parodon nasus[1]	LBP 1135	5635	HQ171429	HQ289714	HQ289137	HQ289328	HQ289521
**Prochilodontidae**							
Prochilodus reticulatus[1]	LBP 6127	29514	HQ171358	HQ289647	HQ289067	HQ289260	HQ289453
Semaprochilodus laticeps[1]	LBP 1383	12728	HQ171245	HQ289536	HQ288955	HQ289152	HQ289343
**Serrasalmidae**							
Catoprion mento[1]	LBP 7556	35624	HQ171392	HQ289679	HQ289100	HQ289293	-
Colossoma macropomum[1]	LBP 5173	26648	HQ171343	HQ289632	HQ289052	HQ289245	HQ289438

Metynnis mola[1]	LBP 667	8050	HQ171433	HQ289718	-	-	HQ289525
Metynnis lippincottianus[1]	LBP 6282	29688	HQ171364	HQ289651	HQ289072	HQ289265	HQ289458
Myloplus rubripinnis[1]	LBP 2184	15570	HQ171252	HQ289543	HQ288962	HQ289159	HQ289350
Mylossoma duriventre[1]	LBP 1823	12921	HQ171248	HQ289539	HQ288958	HQ289155	HQ289346
Piaractus mesopotamicus[1]	LBP 4255	23803	HQ171315	HQ289604	HQ289024	HQ289217	HQ289411
Pygocentrus cariba[1]	LBP 2229	15662	HQ171256	HQ289547	HQ288966	HQ289163	HQ289354
Serrasalmus maculatus[1]	LBP 3698	21836	HQ171285	-	HQ288995	HQ289189	HQ289382
Serrasalmus spilopleura[1]	LBP 3499	20169	HQ171281	HQ289570	HQ288991	HQ289185	HQ289378
Tometes trilobatus[1]	LBP 9072	42585	HQ171425	HQ289711	HQ289133	-	HQ289518
**Triportheidae**							
Agoniates anchovia[1]	LBP 6740	33471	HQ171378	HQ289665	-	-	HQ289472
Agoniates halecinus[1]	LBP 5503	26594	HQ171342	HQ289631	HQ289051	HQ289280	HQ289437
Clupeacharax anchoveoides[1]	LBP 5046	26012	HQ171337	HQ289626	HQ289046	HQ289239	HQ289433
Engraulisoma taeniatum[1]	LBP 4038	22896	HQ171299	HQ289588	HQ289009	-	-
Engraulisoma taeniatum[1]	LBP 4038	22897	HQ171300	HQ289589	HQ289010	-	-
Lignobrycon myersi[1]	LBP 8094	37519	HQ171402	HQ289689	HQ289110	HQ289303	-
Triportheus nematurus[1]	LBP 39	3503	HQ171383	HQ289670	HQ289091	HQ289284	HQ289476
Triportheus orinocensis[1]	LBP 2663	15580	HQ171253	HQ289544	HQ288963	HQ289160	HQ289351
**Cypriniformes**							
Carassius auratus[1]	LBP 9215	43160	HQ171427	HQ289713	HQ289135	-	-
Gyrinocheilus sp.[3]	AMNH 233433		AY788015	AY791399	-	-	AY804074

1- Oliveira *et al.* [1]; 2-Javonillo *et al.* [20]; 3- Calcagnotto *et al.* [19]
