# Supplementary material for: Systematic and historical biogeography of the Bryconidae (Ostariophysi: Characiformes) suggesting a new rearrangement of its genera and an old origin of Mesoamerican ichthyofauna
Source: BMC Evol Biol. 2014 Jul 8;14:152. doi: 10.1186/1471-2148-14-152 (PMC4109779; doi:10.1186/1471-2148-14-152)
Supplement: Additional file 2 — Sequences of primers used in present study. [file 1471-2148-14-152-S2.pdf]

Additional File 2: **Sequences of primers used in present study**.

Gene	Primer name	Primer sequence (5'-3')	Source
16S	16Sa-L	ACGCCTGTTTATCAAAAACAT	[1]
	16Sb-H	CCGGTCTGAACTCAGATCACGT	[1]
Cytb	L14841	AAATCAAAGCATAACACTGAAGATG	[2]
	H15915	CCAATTTGCATGGATGTCTTCTCGG	[3]
	LNF	GACTTGAAAAACCAYCGTTGT	[4]
	H08R2	GCTTTGGGAGTTAGDGGTGGGAGTTAGAATC	[4]
Myh6	F329	CCGCMTGGATGATCTACAC	[5]
1stPCR	A3R1	ATTCTCACCACCATCCAGTTGAA	[5]
Myh6	A3F2	GGAGAATCARTCKGTGCTCATCA	[5]
2ndPCR	A3R2	CTCACCACCATCCAGTTGAACAT	[5]
	Myh6COF1	GACTGTTAACACCAAGAGAGT	[4]
	Myh6COF2	GTTATCCAGTATTTTGCAAGTATTGC	[4]
	Myh6COR1	TTGAACATCTTCTCATACAC	[4]
	Myh6COR2	TTCTCATACACTGACTTAGCCAGTGC	[4]
RAG1	2510F	TGGCCATCCGGGTMAACAC	[6]
1stPCR	4090R	CTGAGTCCTTGTGAGCTTCCATRAAYTT	[6]
RAG1	2535F	AGCCAGTACCATAAGATGTA	[6]
2ndPCR	4078R	TGAGCCTCCATGAACTTCTGAAGRTAYTT	[6]
	Rag1CF1	ACCCTCCGTACTGCTGAGAA	[4]
	Rag1CF2	TACCGCTGAGAAGGAGCTTC	[4]
	Rag1CF3	GAGAAGGAGCTTCTCCCAGG	[4]
	Rag1CF4	GCTTCCATCAGTTTGAGTGG	[4]
	Rag1CF5	CAGCTCTTGGAACATAGGCATCA	[4]
	Rag1CR1	CGTCGGAAGAGCTTGTTGCC	[4]
	Rag1CR2	TGTTGCCAGACTCATTGCCCTC	[4]
	Rag1CR3	CCCTCGCTGGCCCAGGCACC	[4]
	Rag1CR4	ATCTCGTTCCACAATCTCAGGC	[4]
	Rag1CR5	CATGGGCCAGTGTCTTGTGGAGGT	[4]
RAG2	164F	AGCTCAAGCTGCGYGCCAT	[4]
1stPCR	RAG2-R6	TGRTCCARGCAGAAGTACTTG	[7]
RAG2	176R	GYGCCATCTCATTCTCCAACA	[4]
2ndPCR	Rag2Ri	AGAACAAAAGATCATTGCTGGTCGGG	[4]

References

1. Palumbi SR: **Nucleic acids II: the polymerase chain reaction**. In *Molecular Systematics*. Edited by Hillis D, Moritz C, Mable B. Massachusetts: Sinauer Associates Inc.; 1996:205-247.
2. Kocher TD, Thomas WK, Meyer A, Edwards SV, Pääbo S, Villablanca FX, Wilson A: **Dynamics of mitochondrial DNA evolution in animals: ampliWcation and sequencing with conserved primers**. *Proc Natl Acad Sci* 1989, **86**:6196-6200.

3. Irwing DM, Kocher TD, Wilson AC: **Evolution of the cytochrome *b* gene of mammals**. *J Mol Evol* 1991, **32**:128-144.

4. Oliveira C, Avelino GS, Abe KT, Mariguela TC, Benine RC, Ortí G, Vari RP, Castro RMC: **Phylogenetic relationships within the speciose family Characidae (Teleostei: Ostariophysi: Characiformes) based on multilocus analysis and extensive ingroup sampling**. *BMC Evol Biol* 2011, **11:**275.

5. Li C, Ortí G, Zhang G, Lu G: **A practical approach to phylogenomics: The phylogeny of ray-finned fish (Actinopterygii) as a case study**. *BMC Evol Biol* 2007, **7**:44.

6. Li C, Ortí G: **Molecular phylogeny of Clupeiformes (Actinopterygii) inferred from nuclear and mitochondrial DNA sequences**. *Mol Phylogenet Evol* 2007, **44**:386-398.

7. Lovejoy NR, Collette BB: **Phylogenetic relationships of new world needlefishes (Teleostei: Belonidae) and the biogeography of transitions between marine and freshwater habitats**. *Copeia* 2001, 1: 324–338.
